# Pre- and post-operative imaging of cochlear implants: a pictorial review

**DOI:** 10.1186/s13244-020-00902-6

**Published:** 2020-08-15

**Authors:** Gerlig Widmann, Daniel Dejaco, Anna Luger, Joachim Schmutzhard

**Affiliations:** 1 Department of Radiology, Medical University of Innsbruck, Anichstr. 35, A-6020 Innsbruck, Austria; 2 Department of Otorhinolaryngology—Head and Neck Surgery, Medical University of Innsbruck, Innsbruck, Austria

**Keywords:** Cochlear implant, Pre-operative imaging, Post-operative imaging, Electrode array position

## Abstract

Cochlear implants are increasingly used to treat sensorineural hearing disorders in both children and adults. Pre-operative computed tomography and magnetic resonance imaging play a pivotal role in patient selection, to rule out findings that preclude surgery or identify conditions which may have an impact on the surgical procedure. The post-operative position of the electrode array within the cochlea can be reliably identified using cone-beam computed tomography. Recognition of scalar dislocation, cochlear dislocation, electrode fold, and malposition of the electrode array may have important consequences for the patient such as revision surgery or adapted fitting.

## Key points

- Pre-operative imaging plays a pivotal role in patient selection for cochlear implant surgery.
- Key imaging findings on pre-operative scans that may have an impact on the surgical procedure need to be reported.
- Post-operative scans are indispensable to recognize dislocation, fold, and malposition of the electrode array, thereby allowing the best possible management of such a postsurgical complication.

## Introduction

A cochlear implant (CI) is a surgically implanted device consisting of external and internal components [1]. An external microphone and speech processor are worn behind the ear and convert sound into an electric signal. A magnet held external transmitter sends the signal via electromagnetic induction through the skin to an internal receiver–stimulator. The receiver–stimulator converts the signal into rapid electrical impulses which are distributed to multiple electrodes on an electrode array implanted within the cochlea. The electrodes electrically stimulate the spiral ganglion cells along the cochlear turns, which then travel along the auditory nerve axons to the brain for sound perception. Straight lateral wall electrode arrays and pre-curved perimodiolar electrode arrays are available in different lengths for coverage of various cochlear duct lengths.

Over the past few decades, CI surgery has increased and revolutionized the treatment of severe to profound sensorineural hearing loss (SNHL) in both children and adults. In brief, current indications are (a) children (12–24 months) with profound SNHL (> 90 dB) and limited benefit from binaural amplification trial based on the meaningful auditory integration scale; (b) children (2–17 years) with severe to profound SNHL (> 70 dB) with limited benefit from binaural amplification defined by ≤ 20–20% word recognition scores; and (c) adults with moderate to profound SNHL in both ears (> 40 dB) with limited benefit from binaural amplification defined by ≤ 50% sentence recognition in the ear to be implanted (or ≤ 40% by centers for medicare and medicaid services criteria) and ≤ 60% in the contralateral ear or binaurally [1, 2]. More recently, indications have been expanded to patients with single-sided deafness and ipsilateral vestibular schwannoma [2].

Absolute contraindications are complete labyrinthine aplasia, cochlear aplasia, and complete cochlear ossification. CI in cochlear nerve aplasia or hypoplasia is controversial. Patients are less likely to benefit, but meaningful hearing can be achieved in selected cases [3, 4]. In children with syndromic disorders such as CHARGE (congenital features of coloboma of the eye, heart defect, atresia of the nasal choanae, retardation of growth and/or development, genital and/or urinary abnormalities, and ear abnormalities and deafness), CI implant surgery is very challenging due to abnormal anatomy and comorbidity [5, 6]. Disabilities including developmental delay, cerebral palsy, visual impairment, autism, and attention deficit disorder significantly affect the outcomes [7]. For elderly patients, general health problems and life expectancy should be taken into account, and the indications for CI should be considered on a case by case basis [8]. Acute infections such as otitis media and mastoiditis as well as chronic inflammation and cholesteatoma must be adequately controlled before CI surgery [9].

Radiologists play an essential role in the pre- and post-operative evaluation and selection of CI candidates. Pre-operative imaging is essential to diagnose any type of inner ear malformations and to identify other abnormalities in the temporal bone that may be encountered [10, 11]. It allows the best insight into all relevant anatomical details and potential situations which preclude surgery or require modifying standard surgical approaches [12, 13]. Post-operative imaging is important to confirm and document the intended electrode position and to demonstrate any scalar dislocation, cochlear dislocation, electrode fold, or malposition, which can be a possible source of CI malfunction [14].

This pictorial review aims to provide a comprehensive overview of the most relevant pre-operative and post-operative imaging aspects in CI candidates intending to help radiologists and surgeons in routine practice.

## Pre-operative imaging

### Modalities and protocols

Pre-operative imaging in CI candidates is based on high-resolution computed tomography (HRCT) and magnetic resonance imaging (MRI) [15]. Practical generic imaging protocols are given in Table 1. Each modality has its strengths and both modalities are complementary to each other (Table 2) [15–17].

**Table 1:** HRCT and MRI protocols for pre-operative imaging and considerations in children

HRCT	MRI (3T preferred)
120–140 kVp, 100 mAs Helical scan, pitch 0.8–0.9 FOV 15–16 cm 0.5–0.6-mm-slice thickness Sharp kernels, W/L: 4000/500 Slice orientation: axial and coronal	T2w 2-mm-slice thickness, axial, and coronal orientation CISS-3D or vendor-specific equivalent 0.8 slice thickness, axial orientation T1w/T1wC+(contrast-enhanced) fat sat, 2-mm-slice thickness, axial orientation Non-EPI-DWI 2-mm-slice thickness, coronal orientation Contrast media: history of inflammation/infection
Children Sedation +/− Radiation dose +	Children Sedation/general anesthesia + Radiation dose −

**Table 2:** Comparison of CT and MRI in imaging of various pre-operative conditions

	CT	MRI
Bone anatomy	+	−
Membranous labyrinth	−	+
Cochlear sclerosis	+	+
Cochlear fibrosis, inflammation	−	+
Cochlear nerve hypoplasia or aplasia	−	+

**Fig. 1:**
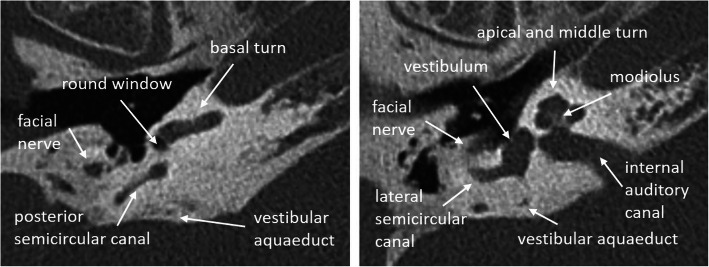
HRCT axial images of normal cochlear anatomy in a 74-year-old man

**Fig. 2:**
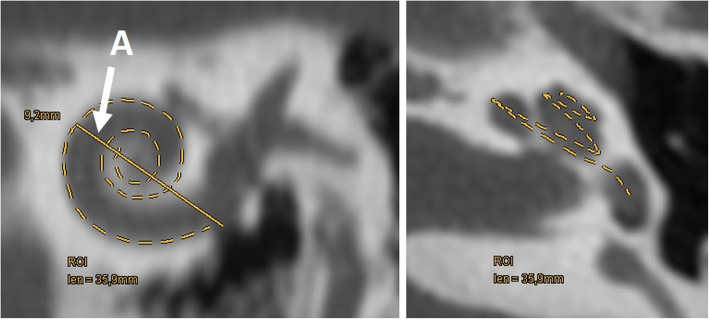
Evaluation of cochlear duct length (CDL) using the formula CDL = 4.16A–2.7 and a 3D segmentation (dashed line). HRCT paracoronal image on the left shows distance A (arrow) from the center of the round window to the far most extension of the basal turn, which measures 9.2 mm. According to the formula the cochlea duct has a length of 35.6 mm. The dashed line in the HRCT paracoronal image and the HRCT paraaxial image on the right shows the 3D segmented cochlear duct which measures 35.9 mm

The strength of HRCT is the detailed visualization of the bony structures of the middle and inner ear. The cochlea is composed of the central modiolus with bony septa that separate the basal, middle, and apical turns (2.5–2.75 turns) (Fig. 1). Each turn contains the scala vestibuli, scala tympani, and the cochlea duct. All relevant anatomical structures including the middle ear, round and oval windows, vestibular aqueduct, segments of the facial nerve, and internal auditory canal are visualized. The length of the cochlear duct (CDL) or two-turn length (2TL) can be three-dimensionally segmented starting from the round window up to the apex or calculated using formulas (see Fig. 2) [18, 19].

Eight different types of cochlear malformations can be differentiated by HRCT: (1) complete labyrinthine aplasia—Michel deformity (complete absence of cochlea, vestibule, vestibular aqueduct, and cochlear aqueduct), (2) cochlear aplasia (absence of the cochlea), (3) rudimentary otocyst (incomplete millimetric otic capsule remnant), (4) common cavity (cochlea and vestibule are represented by a single chamber), (5) incomplete partition of the cochlea (defect in the modiolus and the interscalar septa with three subtypes), (6) cochlear hypoplasia (cochlea with dimensions less than normal with four subtypes), (7) large vestibular aqueduct syndrome (enlarged vestibular aqueduct in the presence of normal cochlea, vestibule, and semicircular canals), and (8) cochlear aperture abnormalities (narrow cochlear nerve canal or internal auditory canal, possibility of an absent, or hypoplastic cochlear nerve) [10].

**Fig. 3:**
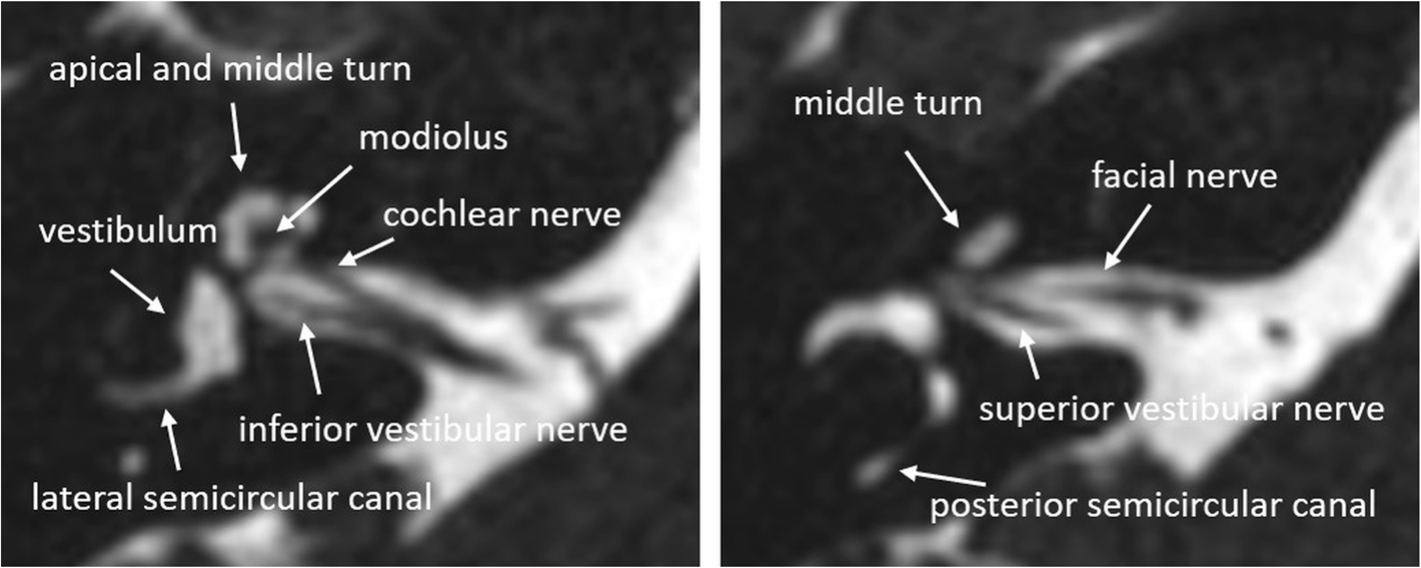
MRI 3D-CISS axial images of normal cochlear anatomy in a 40-year-old woman

The strength of the MRI is the visualization of the fluid content of the membraneous labyrinth (Fig. 3). Visualization of the vestibulocochlear nerve in the fluid-filled internal auditory canal and cerebellopontine angle is only possible by the MRI [16]. History of meningitis, temporal bone fracture, or otosclerosis may lead to cochlear fibrosis or scarring, which appear as a loss of fluid signal. Sclerosis of the cochlea can be seen in HRCT; however, early fibrotic stages may only be depicted by MRI [20]. Contrast enhancement may support the diagnosis of fibrosis and inflammation.

### Key imaging findings which preclude cochlear implantation

**Fig. 4:**
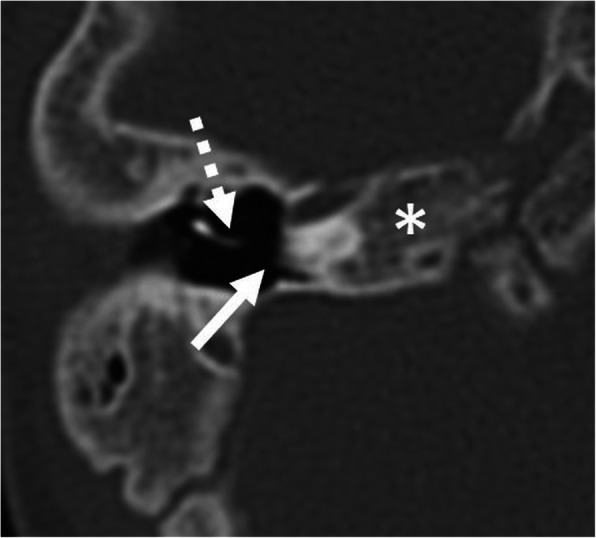
A 1-year-old male patient, with bilateral sensorineural deafness from birth. HRCT axial image shows hypoplastic right petrous bone with a complete absence of the inner ear structures (asterisk), compatible with Michel’s deformity. The medial wall of the middle ear is flat (arrow). Absent round and oval windows. Absent stapes. Normal-looking malleus (dashed arrow)

**Fig. 5:**
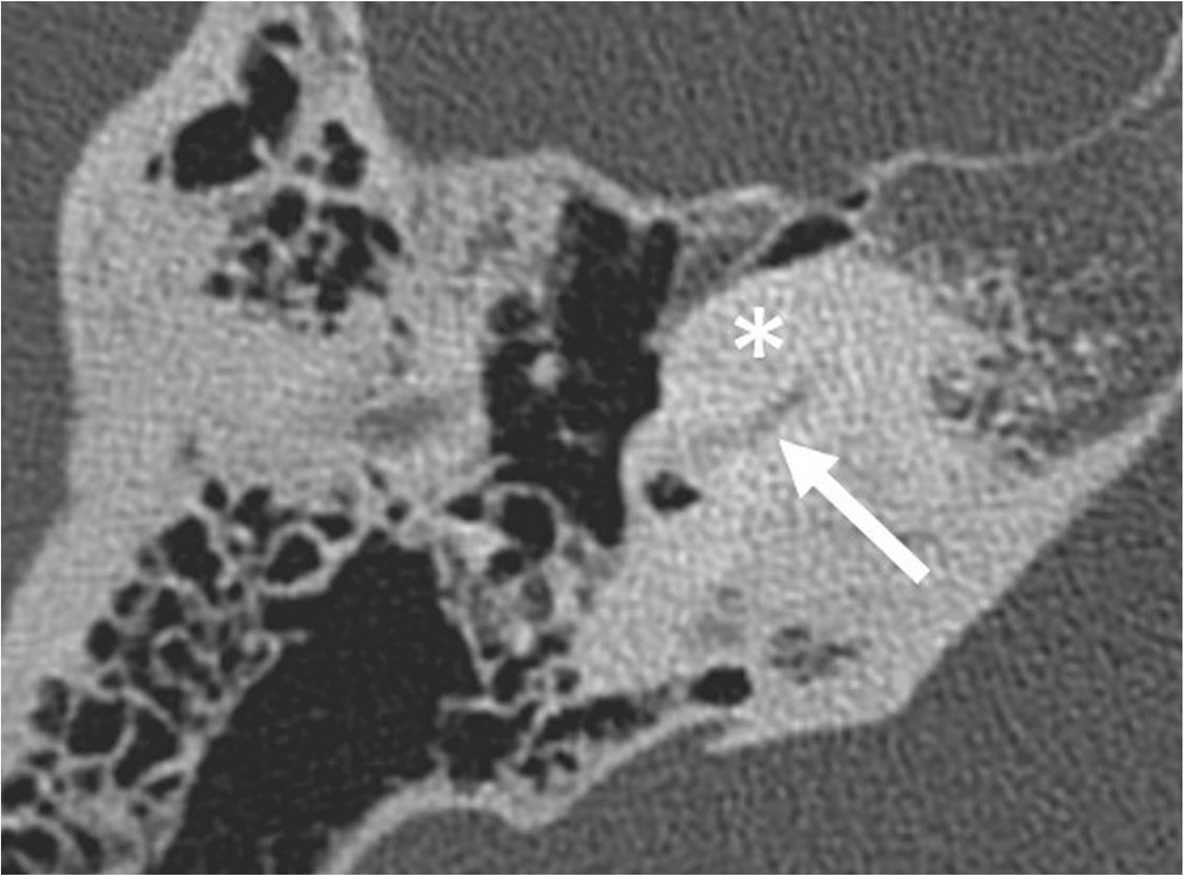
A 38-year-old female patient, with unilateral sensorineural deafness since the age of 12 years. HRCT axial image shows cochlear sclerosis (asterisk). Only a small part of the basal turn of the cochlea can be faintly seen (arrow)

**Fig. 6:**
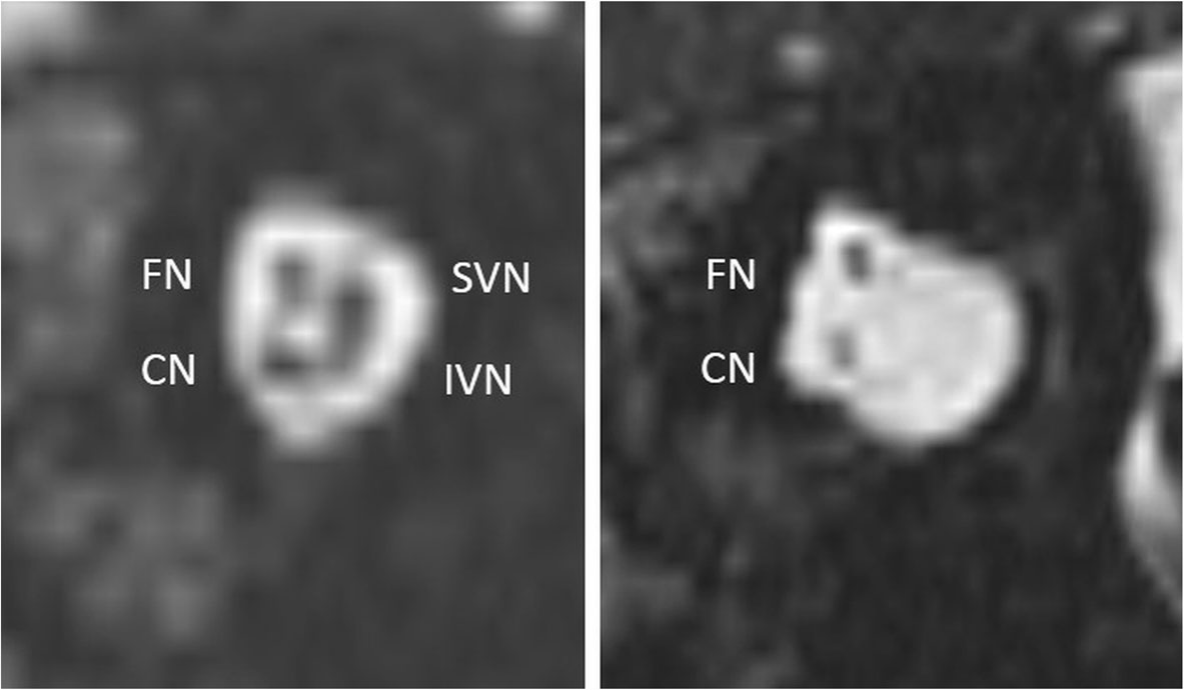
A 57-year-old female patient with unilateral SNHL from birth. MRI 3D-CISS parasagittal image of the internal auditory canal shows regular facial nerve (FN), cochlear nerve (CN), superior vestibular nerve (SVN), and inferior vestibular nerve on the healthy side (left image) and missing SVN and IVN, as well as a hypoplastic CN on the diseased side (right image)

The most commonly accepted imaging findings precluding cochlea implantation are [1]:

- Complete labyrinthine or isolated cochlear aplasia (Fig. 4)
- Cochlear sclerosis (Fig. 5)
- Cochlear nerve deficiency (Fig. 6)

### Key imaging findings which have an impact on the surgical procedure

#### Common cavity malformation

The common cavity is a malformation in which the cochlea and vestibule are represented by a single chamber [10]. It can be exceedingly difficult to place the electrode array close to the neural elements. Satisfactory clinical results are achieved when the stimulating electrode contacts form a loop within the cavity [21–23]. Modification of the cochleostomy shape and looping of the cochlear implant electrode in the implantable cystic space is recommended [22]. Custom-made devices for common cavities are available from some manufacturers [24].

#### Cochlear hypoplasia (Fig. 7)

Cochlear hypoplasia may appear as bud-like cochlea (type I), cystic hypoplastic cochlea (type II), cochlea with less than 2 turns (type III), and with normal basal turn, but severely hypoplastic middle and apical turns (type IV) [10]. Due to the small size of the hypoplastic cochlea, thin and short electrodes are recommended. Thick and long electrodes may not be fully inserted into the cochlea. The cystic hypoplastic cochlea has a potential for cerebrospinal fluid leakage [11].

**Fig. 7:**
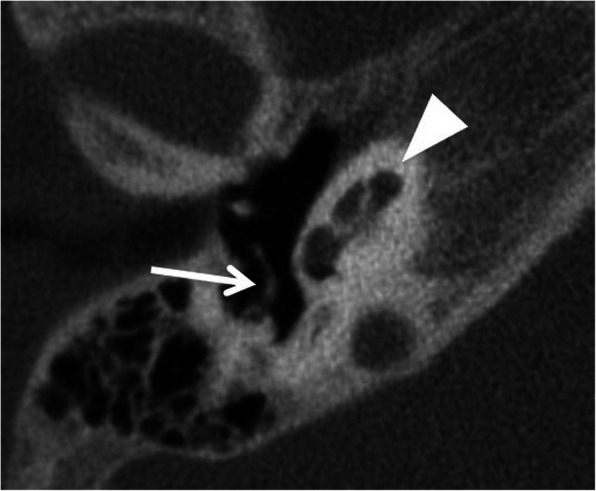
A 5-year-old male patient, with CHARGE syndrome and bilateral severe SNHL from birth. HRCT axial image shows hypoplastic cochlea type III with less than 2 turns (arrowhead). Malformed crus longum incudis and stapes are fused with the posterior tympanic wall (arrow)

#### Incomplete partition (Figs. 8, 9, and 10)

**Fig. 8:**
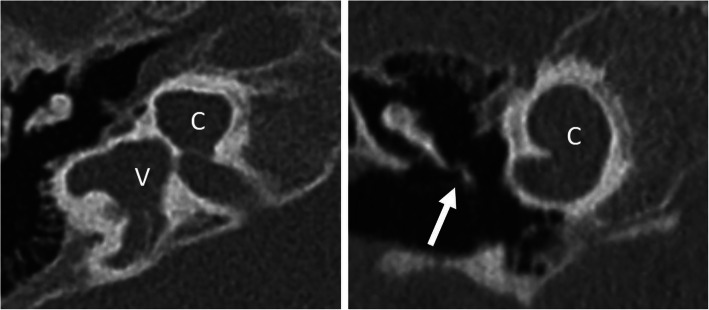
A 1-year-old male patient, with sensorineural deafness from birth. HRCT axial image (left) and coronal image (right) show incomplete partition type I, with empty cystic cochlea (C) and a large dilated vestibulum (V). Stapes is malformed and fused with the incus (arrow)

Incomplete partition type I (Fig. 8) shows no modiolus and interscalar septa [10]. It looks like an empty cystic structure and is accompanied by a large dilated vestibulum. It can be challenging to place the electrode array close to the neural elements [25, 26]. An aggressive attempt at the full insertion of the array may result in misplacement through the deficient modiolus into the internal auditory canal [26].

**Fig. 9:**
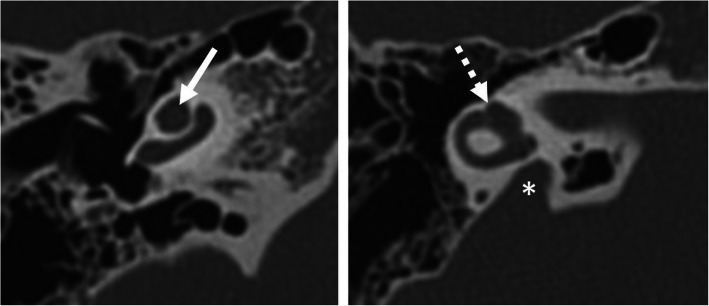
A 12-year-old male patient, with bilateral severe SNLH from birth. HRCT axial images show incomplete partition type II with the cystic apex of the cochlea (arrow) and enlarged vestibular aqueduct (asterisk). Vestibulum is minimally enlarged (dashed arrow), and semicircular canals appear normal

**Fig. 10:**
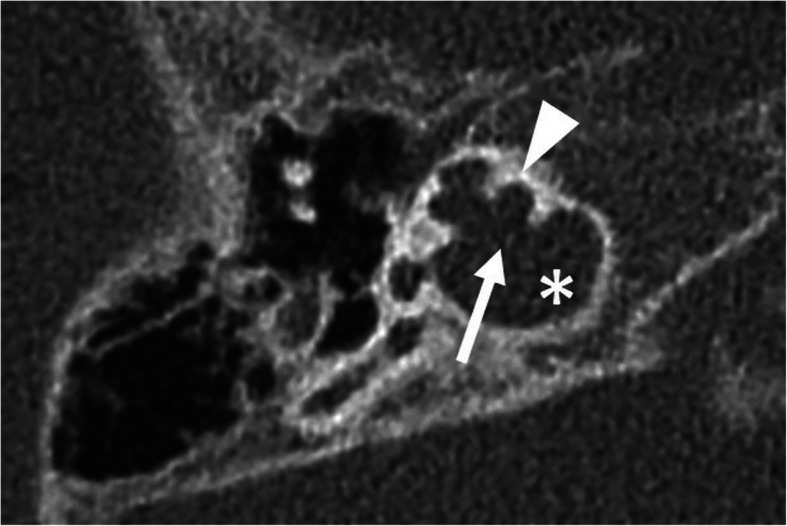
A 1-year-old male patient, with bilateral severe SNLH from birth. HRCT axial image shows incomplete partition type III with empty cochlea with preserved interscalar septa (arrowhead). Modiolus and bony separation of the cochlea and internal auditory canal are absent (arrow). The cochlea is placed directly at the lateral end of the internal auditory canal (asterisk)

Incomplete partition type II (Fig. 9) shows a cystic apex of the cochlea and only the basal parts of the modiolus are present. In addition, the vestibular aqueduct is enlarged, and the vestibule is minimally dilated. The full triad is named Mondini deformity [10].

Incomplete partition type III (Fig. 10) is reported in X-linked deafness [27]. The interscalar septa are present but the modiolus is completely absent. The cochlea is placed directly at the lateral end of the internal auditory canal instead of its usual anterolateral position. The missing bony separation of the cochlea and internal auditory carries an increased risk for an electrode dislocation into the internal auditory canal [28].

Modiolar base defects in incomplete partitions have increased risk of intraoperative cerebrospinal fluid leakage into the middle ear, named gusher [25, 29]. An intraoperative gusher is resulting in a prolonged procedure, hampers the electrode insertion, and increases the risk of meningitis. Tight cochleostomy and thoroughly packing with the tissue around the electrode array may be needed [14]. Dedicated electrodes with a cork stop like electrode design may improve the sealing of the electrode at the cochlear entry [21, 30].

#### Large vestibular aqueduct syndrome (Fig. 11)

This syndrome shows an enlarged vestibular aqueduct with the otherwise regular cochlea, vestibule, and semicircular canals [31, 32]. Cincinnati criteria (midpoint > 0.9 mm or operculum > 1.9 mm) and the Valvassori criterion (midpoint > or = 1.5 mm) for enlarged vestibular aqueduct are used. Cincinnati criteria are found to be more sensitive to identify pediatric cochlear implant patients who might otherwise have no known etiology for their deafness [33]. The enlarged vestibular aqueduct results from an abnormal connection between the perilymphatic and subarachnoid spaces, which transmits a high pressure into the cochlea. This condition may lead to an intraoperative gusher.

**Fig. 11:**
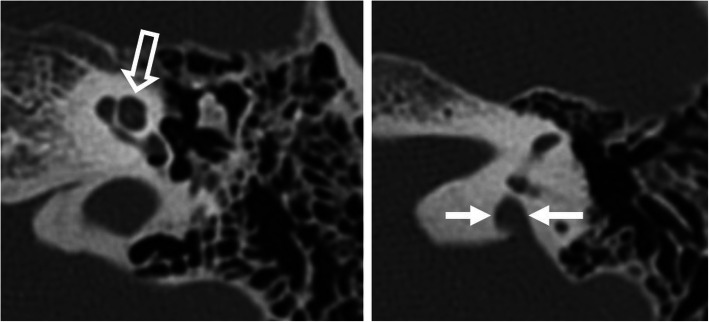
A 56-year-old male patient, with bilateral severe SNLH from birth. HRCT axial images show regular cochlea (open arrow), regular vestibulum and semicircular canals (not shown), and enlarged bony opening for the vestibular aqueduct (arrows), compatible with large vestibular aqueduct syndrome

#### Abnormal position of the facial nerve (Fig. 12)

Malformations of the inner ear are frequently accompanied by abnormal positions of the facial nerve, which increases the risk of facial nerve palsy during surgery [34]. The labyrinthine segment of the facial nerve may show an anterior and superior displacement. The tympanic segment may be superiorly displaced, at the oval foramen, and inferiorly to the oval foramen. The mastoid segment may be lateralized and show a narrow facial recess. Modified surgical approaches such as retrofacial, trans-attic combined with transcanal, or facial recess combined with a transcanal approach are needed [35].

**Fig. 12:**
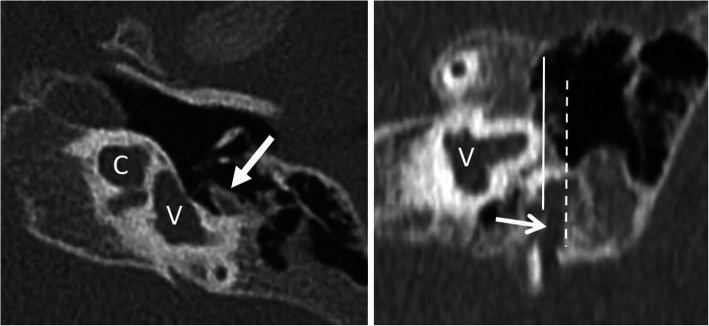
A 1-year-old male patient, with bilateral severe SNHL from birth. HRCT axial image (left image) shows incomplete partition type I with cystic cochlea (C) and vestibulum (V). The horizontal segment of the facial nerve (arrow) is lateralized. HRCT coronal image (right image) shows an interrupted line corresponding to the most lateral aspect of the vertical segment of the facial nerve (open arrow) lateral to the continuous line corresponding to the most lateral bony aspect of the lateral semicircular canal. The normal location of the vertical segment is medial to the lateral semicircular canal

#### Hypoplastic round window or oval window (Fig. 13)

If the cochlear windows cannot be identified, it is very difficult to localize the cochlea for the correct cochleostomy site [5]. A CT-guided approach using a navigation system may be recommended to facilitate surgical orientation [12, 36]. Furthermore, deeply located narrow round windows may provide an awkward angle, which makes it difficult to insert the CI.

**Fig. 13:**
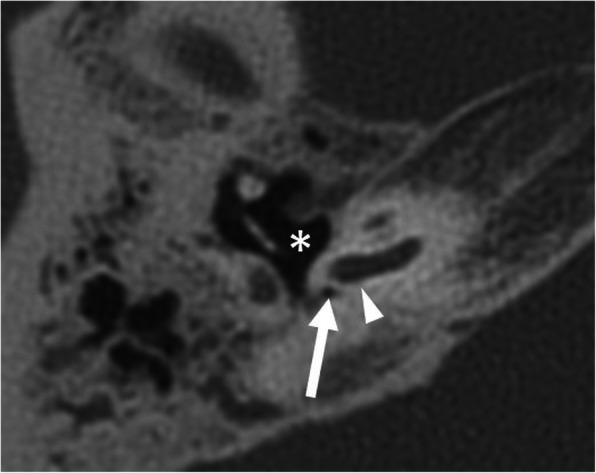
A 65-year-old male patient, with external auditory canal hypoplasia and bilateral SNLH from birth. HRCT axial image shows a hypoplastic round window (arrow) and a small tympanic cavity (asterisk). This condition complicates anatomical orientation and surgical access to the basal turn (arrowhead)

#### Cochlear aperture abnormalities (Fig. 14)

Cochlear nerve canal hypoplasia is defined by stenosis of the bony cochlear nerve canal diameter at the mid-modiolus of 1.5 mm or less [37]. In about one third of cases of stenotic bony cochlear nerve canal, there is also a stenotic internal auditory canal with a diameter at the midpoint of the canal smaller than 2.5 mm. These patients frequently show a hypoplastic or aplastic cochlear nerve, which impedes clinical outcomes [38]. Hypoplasia of the vestibulocochlear nerve may be seen in cochlear aplasia, complete aplasia of the semicircular canals, severe cochlear hypoplasia, common cavity, incomplete partition type 1, and mild cochlear hypoplasia, with decreasing degrees of correlation [39].

**Fig. 14:**
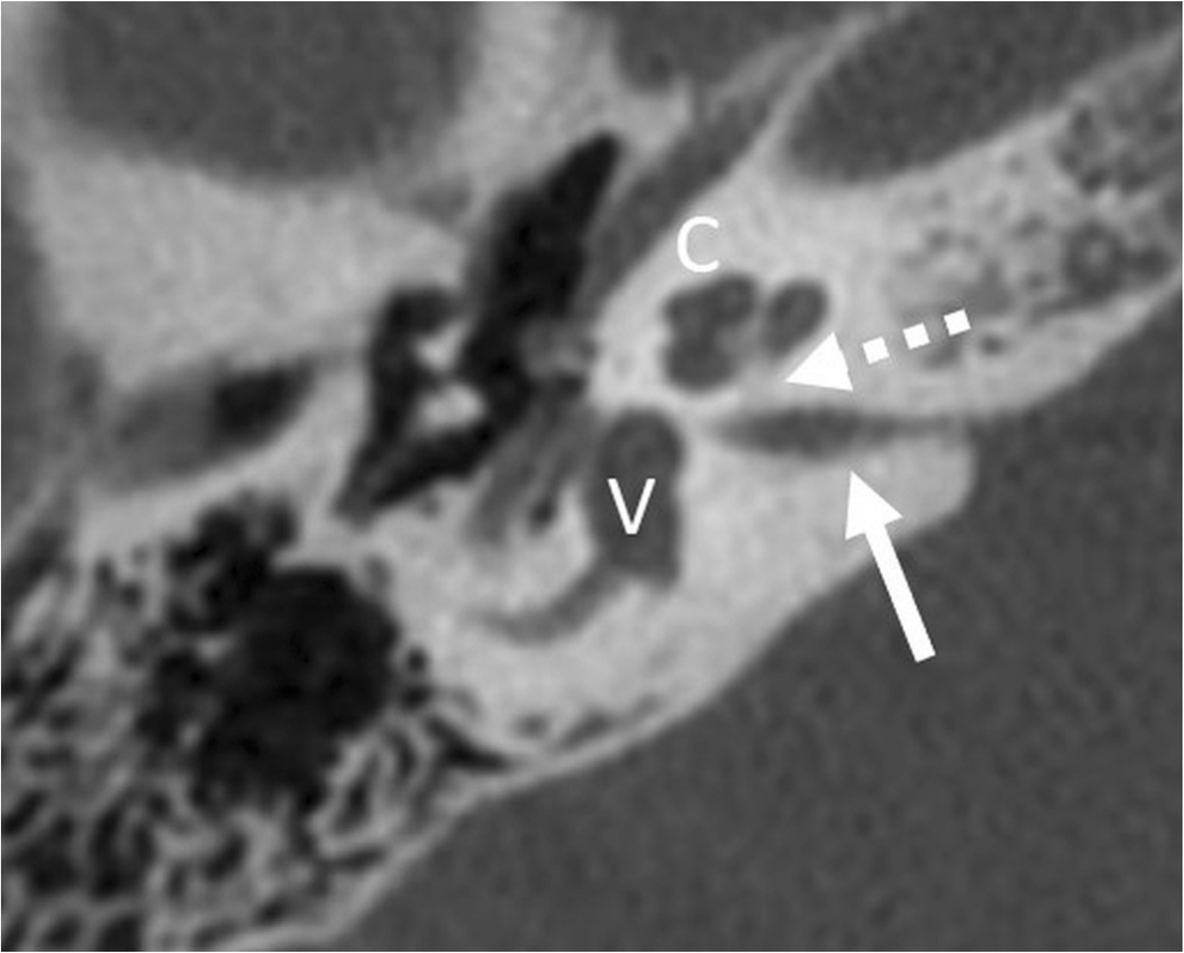
A 16-year-old female patient, with unilateral deafness from birth. HRCT axial image shows high-grade stenotic bony cochlear nerve canal (dashed arrow) and the stenotic internal auditory canal (arrow). Cochlea (C) and vestibulum (V) appear normal

#### Cochlear fibrosis (Fig. 15)

Chronic otitis media, temporal bone fractures, meningitis, and Cogan’s syndrome may lead to cochlear fibrosis [40]. In some cases, no obvious cause can be found. Even without evidence of sclerosis in CT, dense fibrotic tissue may pose a significant problem as the electrode may not be inserted into the cochlea. Surgical modifications including subtotal petrosectomy, split electrode arrays, and inverse approaches can be valuable options [36].

**Fig. 15:**
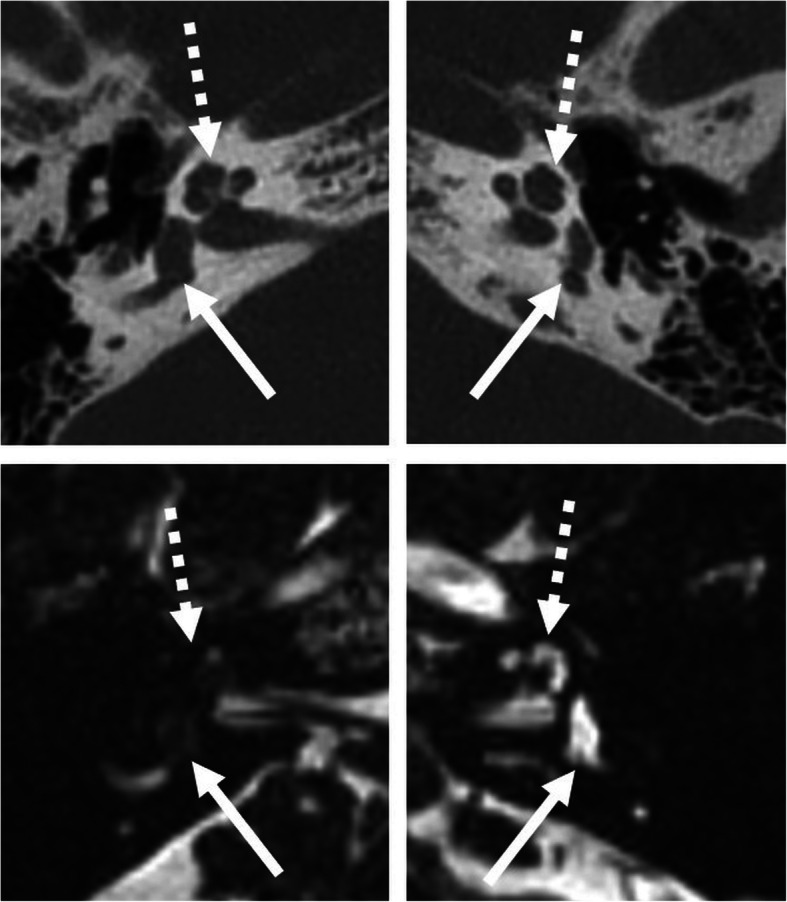
A 51-year-old male patient, with unilateral SNHL from several years. HRCT axial images (upper image) shows normal bony cochlea (dashed arrow) and vestibulum (arrow) on both sides. However, MRI 3D-CISS axial images (lower images) show loss of signal intensity of the right cochlea (dashed arrow) and vestibulum (arrow), compatible with fibrosis in early-stage labyrinthitis ossificans

#### Otosclerosis (Fig. 16)

Far advanced otosclerosis may show irregular ossifications affecting the cochlea, which prevent regular entry and insertion of the CI. Split electrode arrays and inverse approaches can be successful [36].

**Fig. 16:**
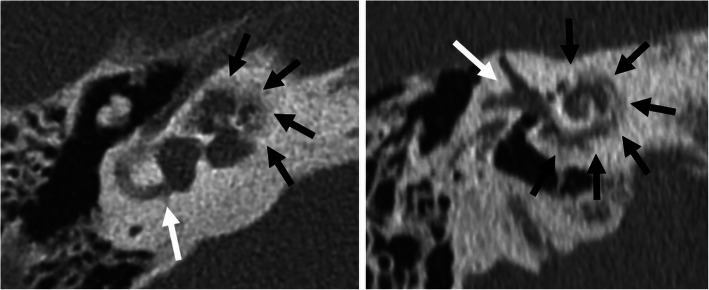
A 70-year-old male patient, with progressive SNHL from advanced otosclerosis. HRCT axial (left image) and paracoronal (right image) image show irregular ossifications affecting the cochlea (black arrows). Vestibulum and semicircular canals (white arrows) are not affected

#### Chronic otitis media and cholesteatoma (Fig. 17)

Identification of a chronic otitis media with or without cholesteatoma is important to prevent infection of the labyrinth during surgery [41]. Either a single-stage surgery with myringoplasty and thereafter the cochlear implantation is performed in the same procedure, or a more than one-staged surgery—firstly eliminating any disease, performing a myringoplasty or tympanoplasty and then performing the cochlear implantation 3–6 months later—is required. Such patients carry a mildly increased risk of device explantation, particularly in open cavity procedures [42].

**Fig. 17:**
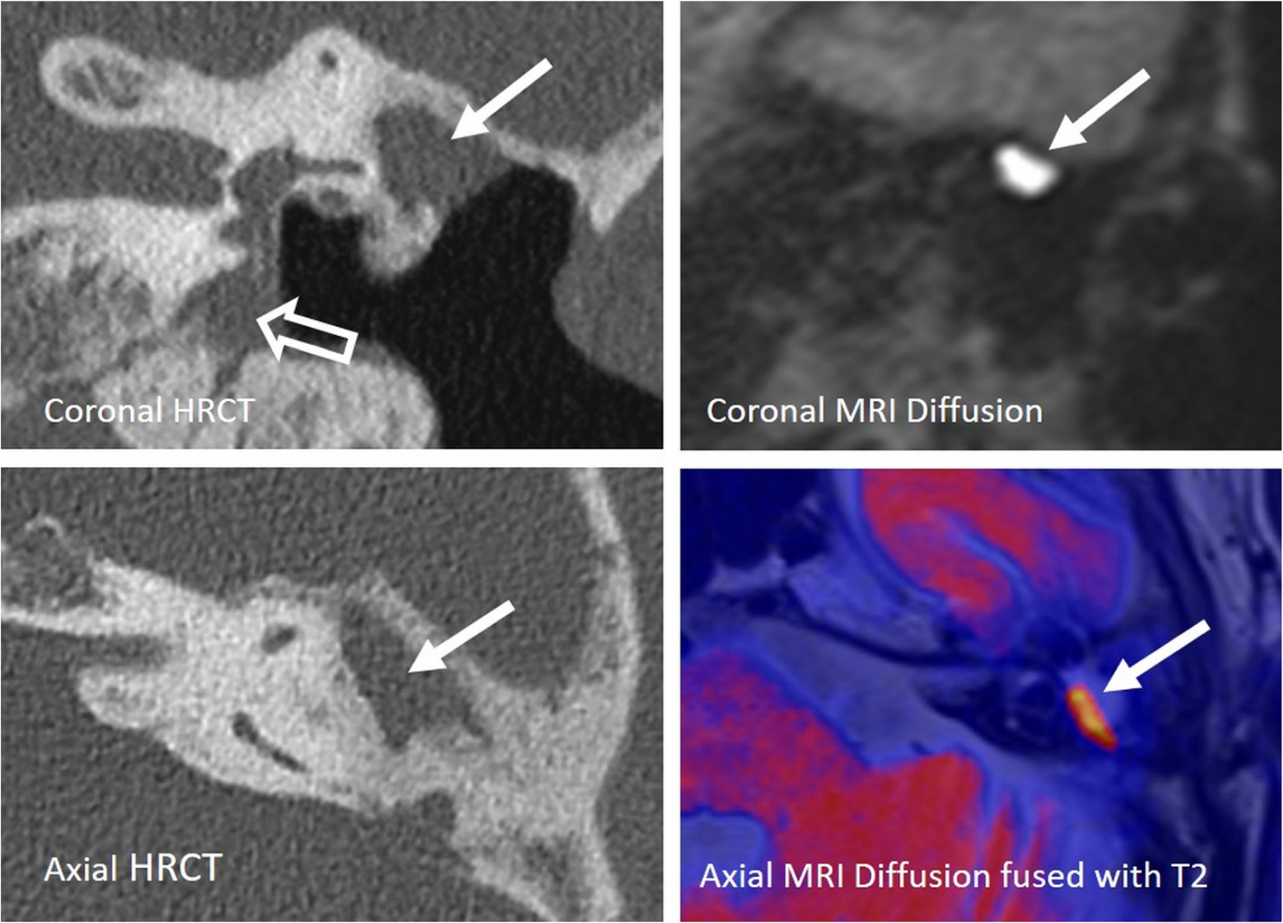
A 80-year-old male patient, with severe SNHL enrolled for cochlear implant surgery. The patient had chronic middle ear infections and cholesteatoma surgery with tympanoplasty type IV several years ago. HRCT coronal and axial images (left images) show opacified antrum (arrow) and opacified hypotympanon (open arrow). A recurrent cholesteatoma cannot be ruled out. Corresponding MRI diffusion coronal image and color-coded axial image fused with T2 (right images) show a high signal intensity in the opacified antrum, typical for cholesteatoma (arrow). The diagnosis was surgically verified, and the patient successfully received a CI one month after revision surgery

## Post-operative imaging

### Modalities and protocols used to assess cochlear implants

Post-operative imaging is required when a malfunction of the device is suspected [43]. However, the authors perform—and recommend to do so—a post-operative examination in every patient to confirm the correct position of the implant electrode. Information regarding basal electrode location helps improving programming accuracy, associated frequency allocation, and audibility with appropriate deactivation of extracochlear electrodes [44].

**Fig. 18:**
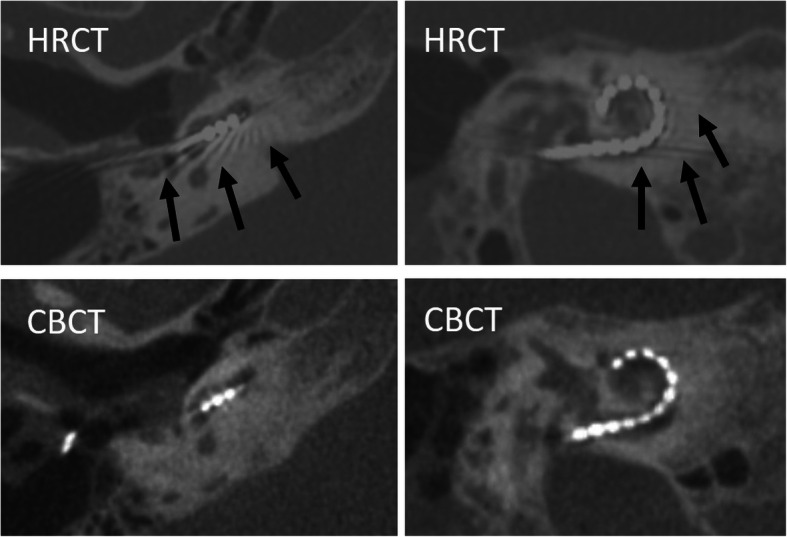
Comparison of postoperative HRCT and CBCT in a 53-year-old female patient. Axial (left images) and paracoronal (right images) images. HRCT images show more metal artifacts (arrows) and blooming artifacts than CBCT images. However, the scalar location and number of electrode contacts can be reliably documented in both modalities

The post-operative position of the electrode array is evaluated using HRCT (similar protocol as for pre-operative imaging) or cone-beam computed tomography (CBCT) (Fig. 18). CBCT has a higher spatial resolution but the performance of different models of CBCT may vary. In general, CBCT is associated with lower dose and less metal artifacts when compared to HRCT (see Table 3 and Fig. 18) [45]. The scalar location of the electrode array can be identified by CBCT with a sensitivity of 100% and specificity of 90% [46]. The last generation CT scanners have significant dose saving options and provide iterative reconstructions for metal artifact reduction, which may reduce differences between both modalities [47, 48]. Conventional X-rays including Stenvers projection cannot inform about the scalar location and may be difficult to interpret, especially in the case of malposition [49].

**Table 3:** Comparison of HRCT and CBCT in evaluation of cochlea implants

	HRCT	CBCT
Radiation dose	+	+/−
Metal artifacts	+	−
Electrode contacts	+/−	+
Scalar localization	+/−	+

**Fig. 19:**
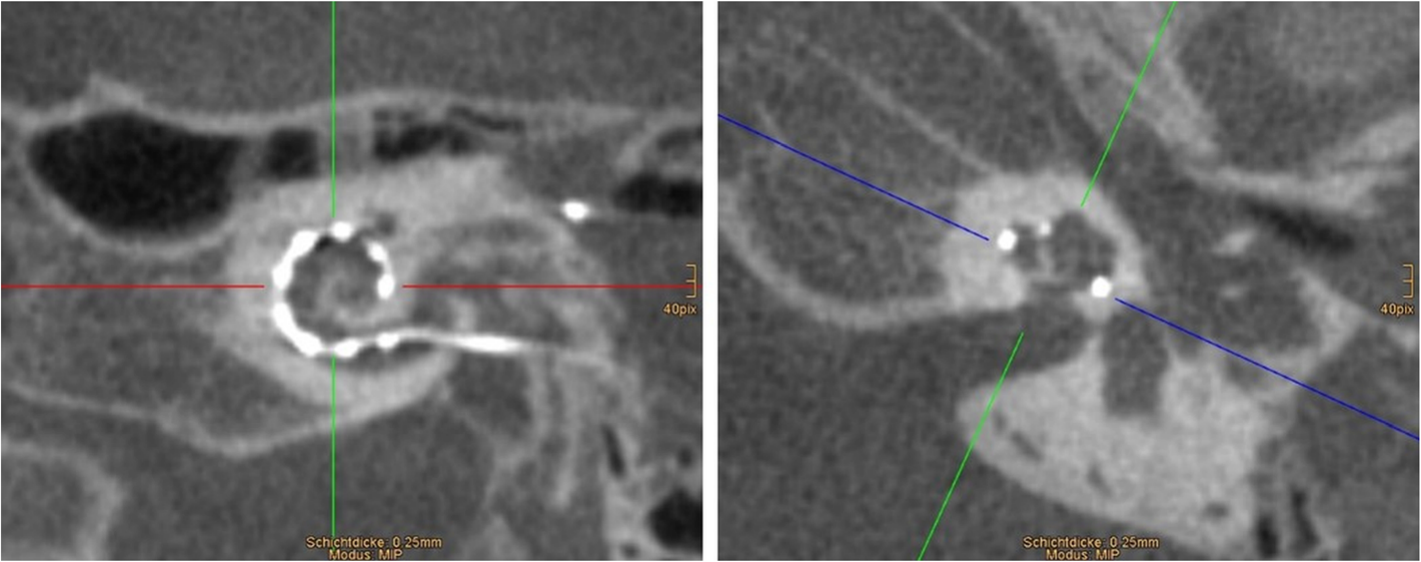
Postoperative evaluation of cochlear implant location using multiplanar mid-modiolar reconstructions. CBCT paracoronal reconstruction at the basal turn (left image) and paraaxial reconstruction through the modiolus (right image). Maximum intensity projections may be used to visualize the entire electrode array

The visibility of the electrode array depends on the size and spacing of electrode contacts. An extensive overview of the cochlear implant electrode array designs from different manufacturers can be found in the article by Dhanasingh et al. [21]. For optimal evaluation of the electrode, a paraxial mid-modiolar plane is selected using multiplanar reconstructions (Fig. 19). Maximum intensity projections with variable slice thickness can be used for the entire visualization of the electrode array. The insertion depth of the cochlear implant can be given as the radial position of the tip ranging from 45° to a theoretical maximum of 900° (full two and a half turns) [50].

### Normal post-operative imaging findings

#### Regular electrode (Fig. 20)

Depending on the anatomical situation, the electrode array is inserted into the cochlea via three routes: (a) round window (preferred), (b) extended round window—enlarging and then opening the round window by drilling the anterior-inferior margin, and (c) cochleostomy—surgical opening of the cochlea [51]. The electrode array is intended to be placed in the scala tympani with close contact to the organ of Corti [1, 52]. This placement may provide the best audiologic outcomes with an excellent speech perception and high rates of hearing preservation [53]. Depending on the type of implant and length of the cochlea, the location of the first electrode contact may be located 3–4 mm from the round window opening [21]. Periomodiolar electrode arrays may be located more closely to the modiolus than straight lateral wall electrodes.

**Fig. 20:**
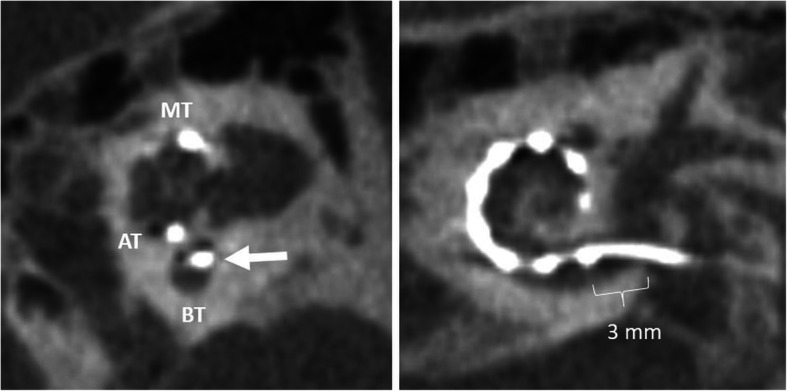
A 15-year-old male patient, with normal postoperative finding after CI surgery. CBCT paracoronal image (left image) shows an electrode array located in the scala tympani (lower segment) of the cochlear duct (white arrow). BT – basal turn. MT – middle turn. AT – apical turn. CBCT paraaxial image (right image) shows the most basal electrode contact for this type of implant correctly located 3 mm below the round window

#### Split electrode (Fig. 21)

Split electrodes are two electrodes which are independently implanted in the basal and the second cochlear turns. Such an approach may be needed in patients with a cochlea ossification/fibrosis and in far-advanced otosclerosis [36, 54].

**Fig. 21:**
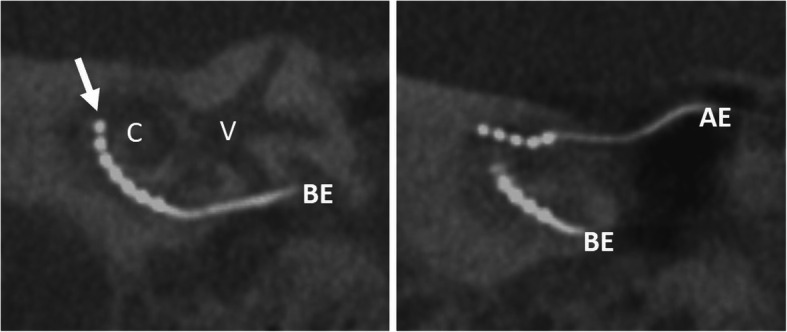
A 70-year-old male patient, with normal postoperative finding after split electrode surgery due to far advanced otosclerosis. CBCT paracoronal image (left) shows basal electrode (BE) with the tip up to the first half of the basal turn (arrow). CBCT paraaxial maximum intensity projection image shows the additional apical electrode (AE) in the middle turn. C – cochlea. V – vestibulum

#### Retrograde electrode (Fig. 22)

Postmeningitic basal turn ossification and fibrosis may block successful antegrade cochlear implantation despite the availability of sophisticated implants and advanced drill-out procedures. In such a case, a retrograde electrode insertion through a cochleostomy near the apex can be performed [55].

**Fig. 22:**
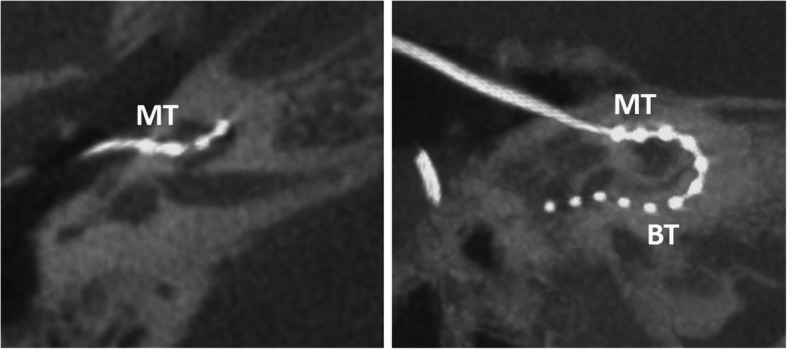
A 51-year-old male patient, with normal postoperative finding after retrograde electrode surgery due to cochlear fibrosis. CBCT axial image (left image) shows electrode entering at the middle turn (MT). CBCT paraaxial maximum intensity projection image (right image) shows the electrode running down the basal turn (BT). The two most distal electrode contacts are in the tympanic space and need to be deactivated

### Unfavorable positions and immediate complications

Complications from cochlear electrode insertion are related to the degree of damage to the organ of Corti located at the basilar membrane and damage of neuronal structures at the spiral lamina [56]. Histological evaluation classifies different grades of electrode-induced trauma: lifting of the basilar membrane (grade 1), damage of the spiral ligament (grade 2), electrode translocation from the scala tympani to the scala vestibuli (grade 3), and fracture of the osseous spiral lamina or modiolus (grade 4) [57].

#### Lifting of the basilar membrane (Fig. 23)

An electrode array located in an intermediate position close to the midline of the cochlear lumen elevates the basilar membrane and bends or deforms the spiral ligament (grade 1 trauma) [58, 59]. It is more frequently observed using lateral wall electrodes compared with perimodiolar electrodes [60]. Damage to both the lateral cochlea wall and osseous spiral lamina tend to cause the new bone formation and fibrous tissue within scala tympani, which may result in a later reduction of the hearing performance [61].

**Fig. 23:**
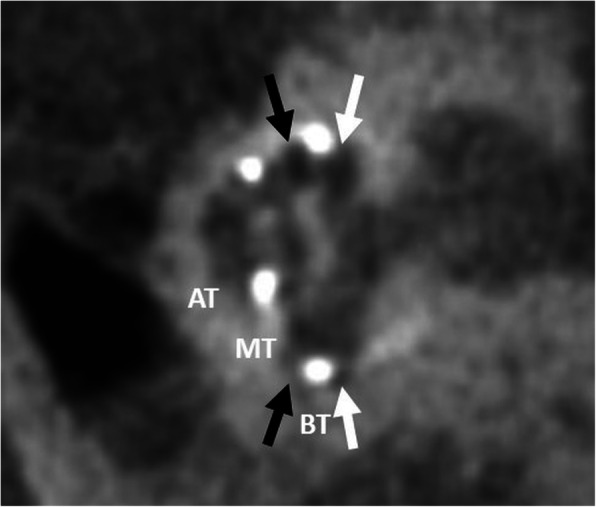
A 72-year-old male patient, with electrode lifting the basilar membrane. CBCT paracoronal image shows the electrode array located in a lateralized and elevated intermediate position between scala vestibuli (black arrow) and scala tympani (white arrow). BT – basal turn. MT – middle turn. AT – apical turn

#### Scala vestibuli (Fig. 24)

An electrode array placed in the scala vestibuli is more frequently seen after a cochleostomy approach [53]. This condition may show an increased risk of damage to sensorineural structures and spiral ganglia which may result in a less favorable outcome.

**Fig. 24:**
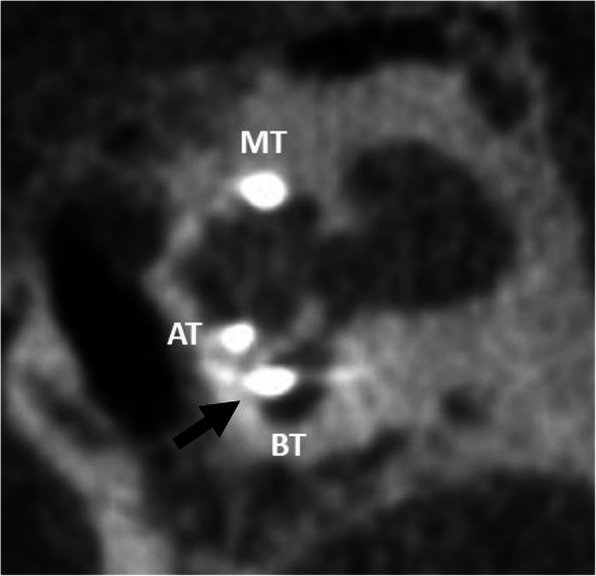
A 70-year-old female patient, with an electrode placed in the scala vestibuli. CBCT paracoronal image shows an electrode array located in the scala vestibuli (upper segment) of the cochlear duct (black arrow). BT – basal turn. MT – middle turn. AT – apical turn

#### Scalar translocation (Fig. 25)

Electrode array translocation from scala tympani into scala vestibuli may be seen in > 20% and is more frequently observed when pre-curved electrodes are used [50, 62, 63]. Translocation usually occurs at 45–180° insertion depth. It leads to a basilar membrane injury which may induce hearing loss. In a retrospective analysis of 63 patients, scalar translocation has been associated with an increase of the necessary stimulus charge [50].

**Fig. 25:**
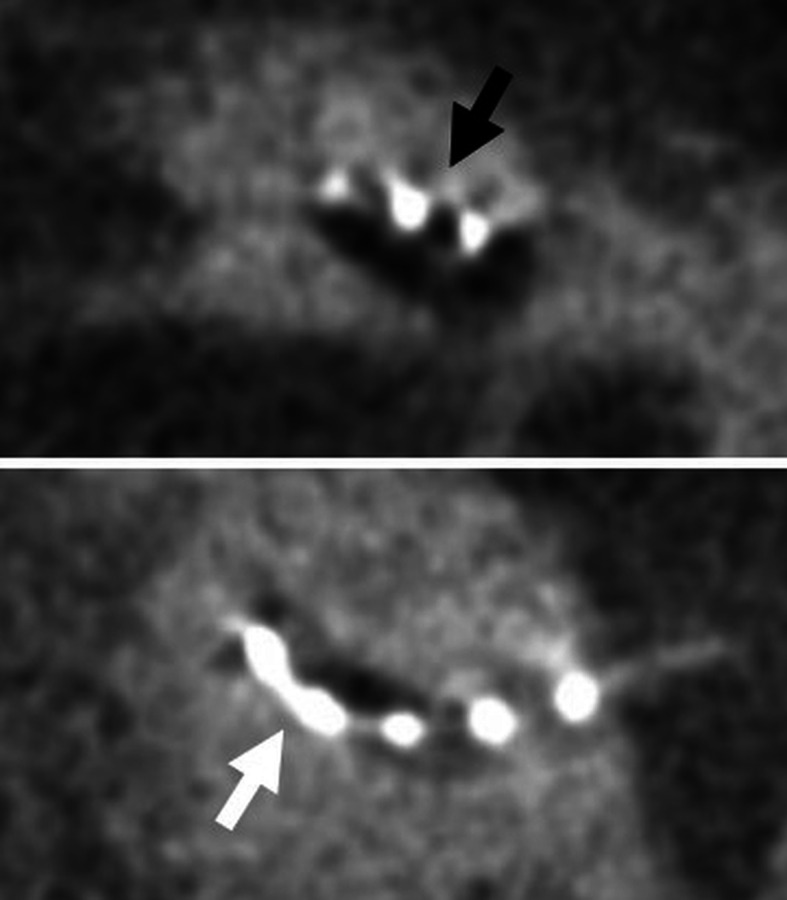
A 62-year-old male patient, with the scalar translocated electrode. CBCT axial images show translocation of the electrode array from scala tympani (white arrow) into scala vestibuli (black arrow). The electrode was inserted via cochleostomy

#### Overinsertion (Fig. 26)

An overinserted electrode array can be recognized when the most proximal contact of the electrode is located more than 3–4 mm from the round window/cochleostomy. This may occur when the opening of the round window or cochleostomy is too large and the electrode is pushed too far into the cochlea. Clinical consequences can be reduced stimulation of the high frequencies, which may result in a poorer speech understanding [21].

**Fig. 26:**
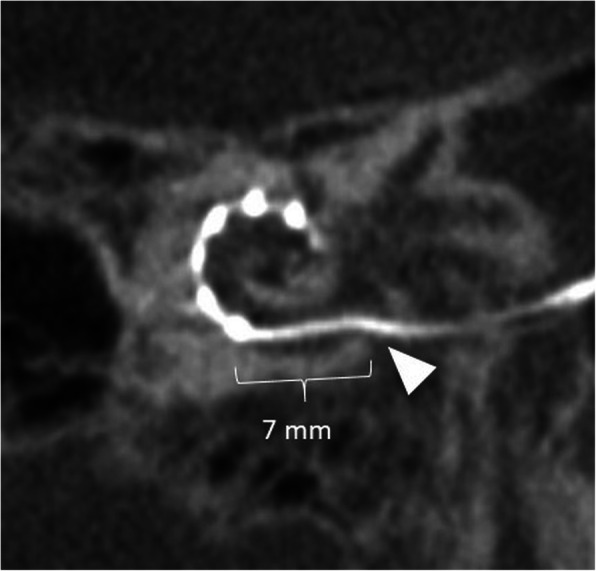
A 11-year-old male patient, with overinserted electrode. CBCT paraaxial maximum intensity projection image shows the most basal electrode contact 7 mm from the round window (arrowhead)

#### Underinsertion (Fig. 27)

An underinserted electrode array exposes several contacts of the electrode array outside of the cochlea. The electrode array has been chosen too long or extruded contacts were a clinical compromise. Risk factors are otosclerosis, meningitis, chronic otitis media, temporal bone fractures, and neurofibromatosis-2 [64]. Underinsertion may result in a functional impairment because the external electrode contacts will not be able to stimulate the spiral ganglion cells. If revision surgery is needed, it should be performed within days, before healing and scaring processes have set in, for easier accessibility.

**Fig. 27:**
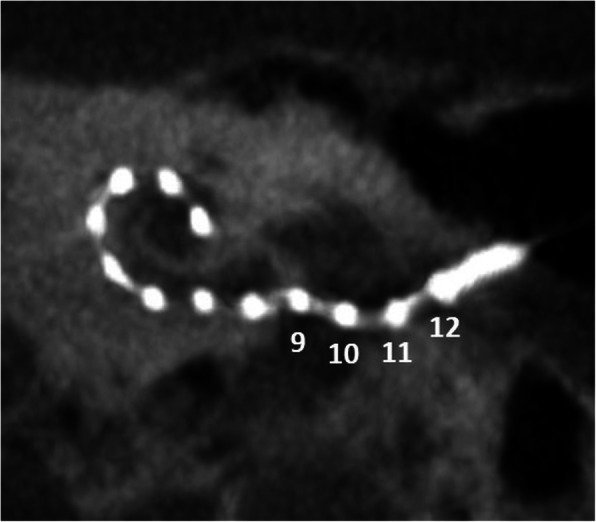
A 66-year-old female patient, with underinserted electrode. CBCT paraaxial maximum intensity projection image shows extracochlear location of electrode contacts 9 to 12

#### Electrode pinching (Fig. 28)

When the electrode array is inserted with too much force or cannot pass further into the cochlea duct, a bending or a more severe accordion-like pinching of the electrode array may be observed [36, 65]. The more severe the bending, the more likely is a mechanical damage of the electrode array. If electrodes overlap, they need to be deactivated.

**Fig. 28.**
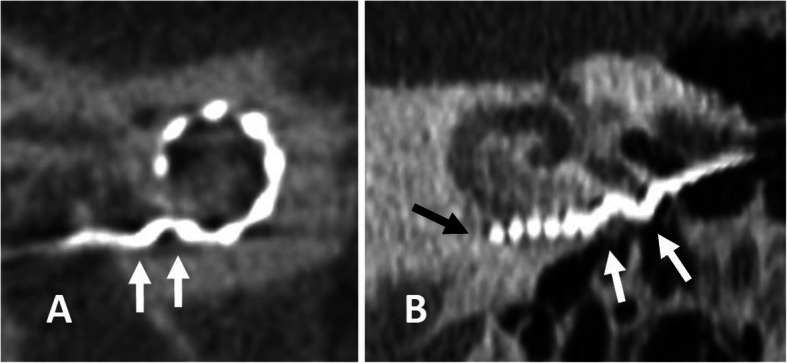
**a** A 75-year-old female patient, with electrode bending. CBCT paraaxial image shows electrode bending (arrows) at the basal turn. **b** A 70-year-old male patient with electrode pinching in far advanced otosclerosis. CBCT paraaxial image shows accordion-like pinching of the basal parts of the electrode array (white arrows). The tip of the electrode array sticks at the basal turn and does not turn around the modiolus (black arrow)

#### Tip fold-over (Fig. 29)

Tip fold-over may have an occurrence rate of 1.5% and usually occurs with flexible and slim perimodiolar electrodes [63, 66, 67]. Fold-over in the cochlea may lead to the rupture of the basilar membrane. Overlapping electrode contacts may need to be deactivated [68]. This may improve performance and avoid revision surgery.

**Fig. 29:**
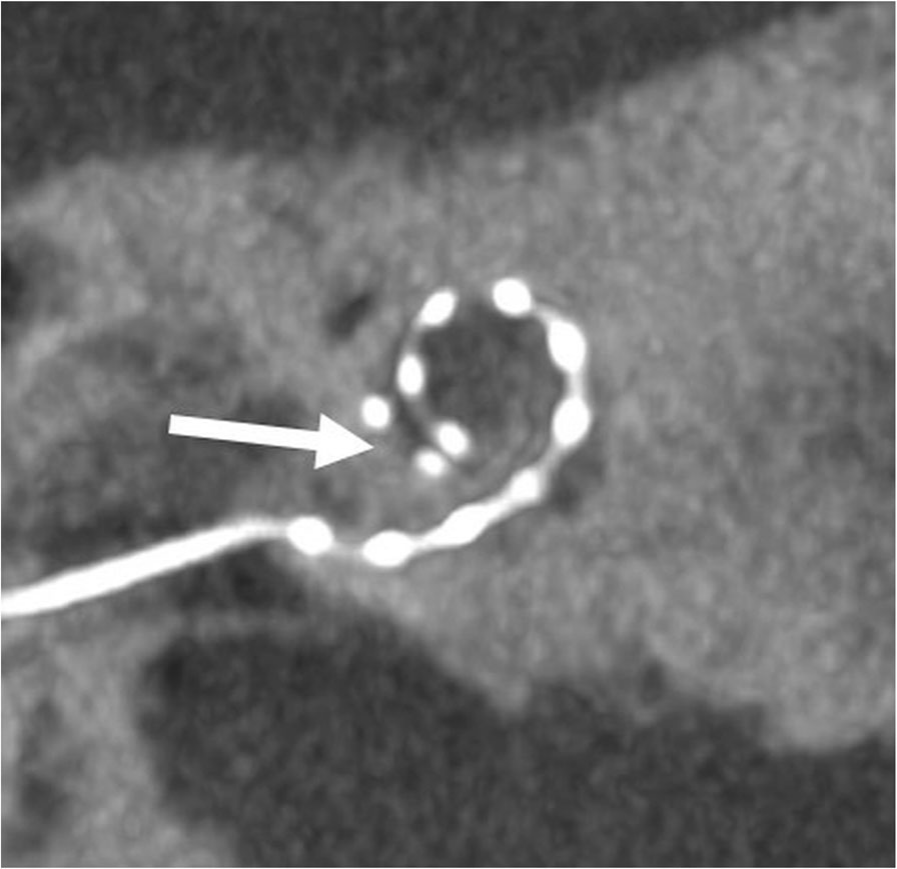
A 66-year-old male patient, with tip fold-over. CBCT paraaxial maximum intensity projection image shows fold-over of the tip of the electrode in the cochlea (arrow)

#### Basal fold-over (Fig. 30)

When the electrode array cannot be fully inserted, excessive pushing may result in a fold-over of the basal part of the electrode array. Extensive fold-over may present as a second electrode array in the basal turn [59, 63]. The basilar membrane may often be ruptured.

**Fig. 30:**
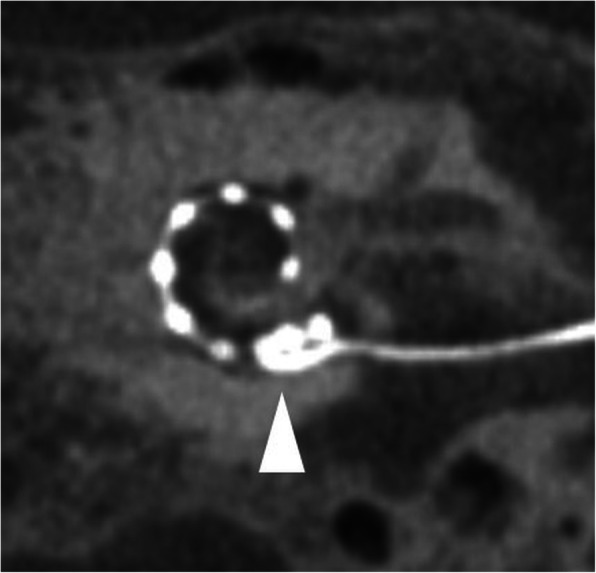
A 55-year-old female patient, with basal fold-over. CBCT paraaxial maximum intensity projection image shows fold-over of the basal part of the electrode within the cochlea (arrowhead)

#### Malposition in the tympanic cavity (Fig. 31)

It the condition of an angulated entry to the basal turn, e.g., in case of CHARGE syndrome, the electrode array may not enter the basal turn and remains in the tympanic cavity near the round window niche [5]. Revision surgery for extracochlear electrode malposition should be performed by experienced surgeons.

**Fig. 31:**
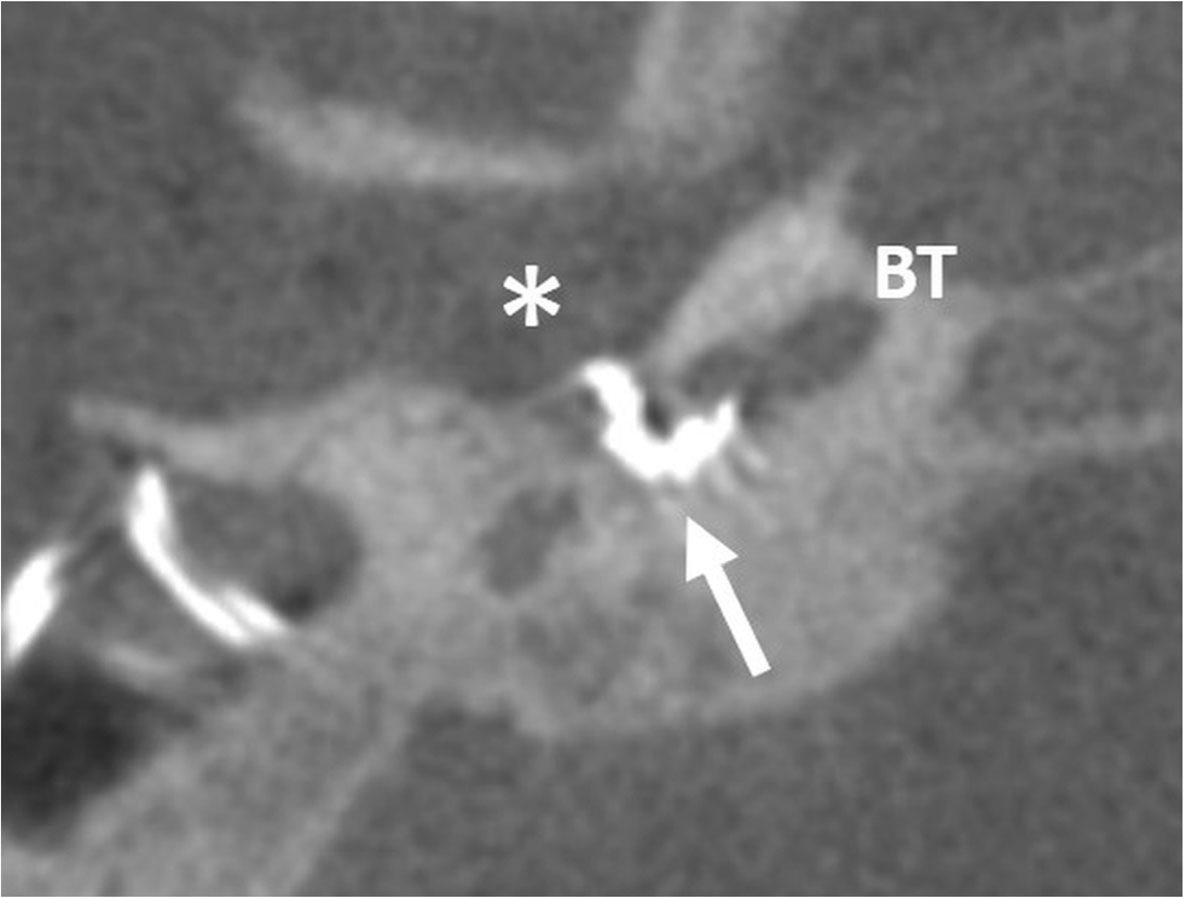
A 13-year-old female patient, with CHARGE syndrome and malposition of the electrode in the tympanic cavity (asterisk). CBCT axial image shows the apical part of the electrode array (arrow) not passing the angled entry of the round window into the basal turn (BT)

#### Malposition in the internal auditory canal (Fig. 32)

The electrode array enters into the internal auditory canal or may form a more basal slope within the internal auditory canal. The condition of an incomplete partition type III is a risk factor, since there is no bony separation between cochlea and internal auditory canal [69, 70].

**Fig. 32:**
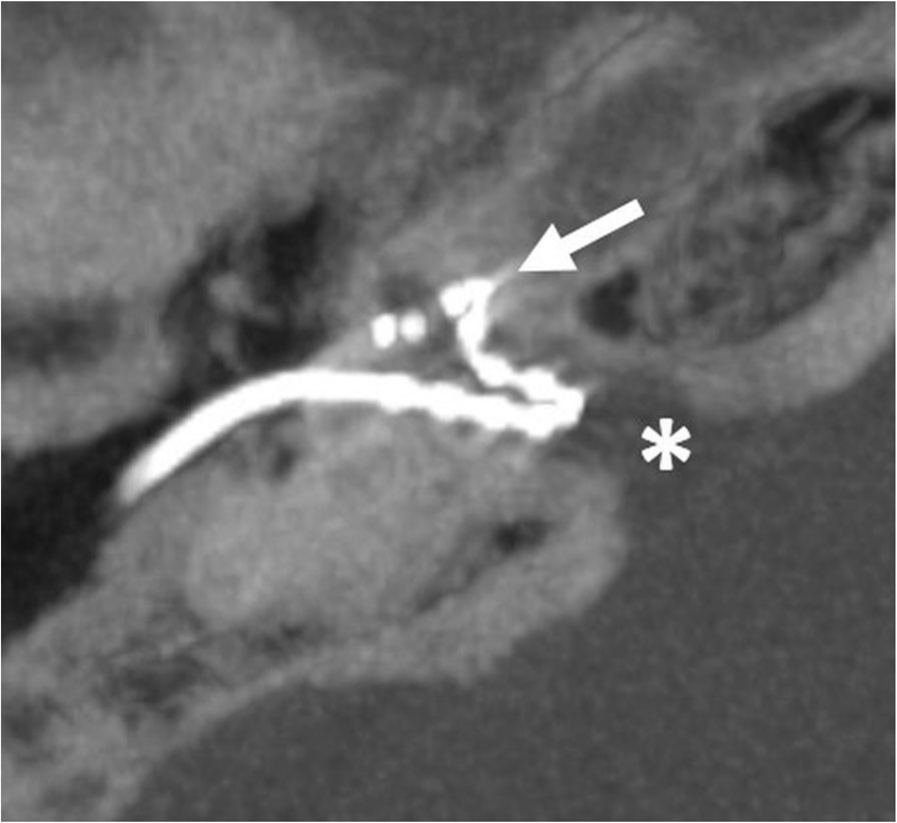
A 20-year-old male patient, with incomplete partition type III and malposition of the electrode in the internal auditory canal. CBCT paraaxial maximum intensity projection image shows part of the electrode array in the basal turn (arrow), but the rest of the electrode forming a slope within the internal auditory canal (asterisk)

#### Malposition in vestibulum and semi-circular canals (Fig. 33)

The electrode array may enter the vestibulum or semi-circular canals in small round windows in children, or, in the condition of an anteriorized facial nerve, because there is an awkward insertion angle for the electrode array via the retro-facial route [71].

**Fig. 33:**
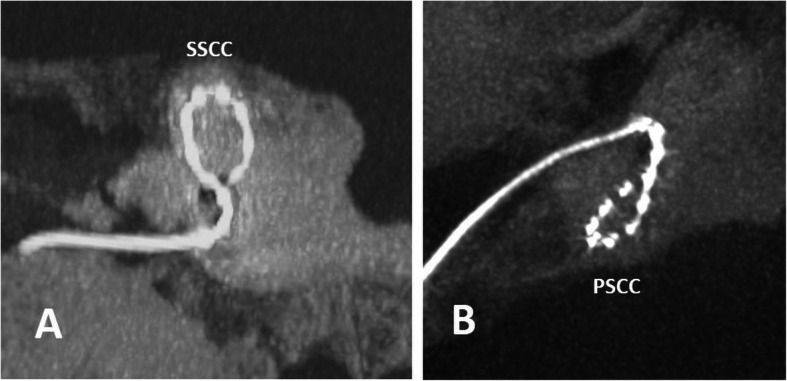
**a** A 86-year-old male patient, with immediate postoperative nausea. CBCT paracoronal maximum intensity projection image shows the malposition of the electrode in the superior semi-circular canal (SSCC). **b** A 20-year-old male patient with asymptomatic malposition of the electrode in the posterior semi-circular canal (PSCC) on CBCT paraaxial maximum intensity projection image

#### Canal of the internal carotid artery or Eustachian tube

Extracochlear electrode array placements like placement in the canal of the internal carotid artery or the Eustachian tube have been very rarely reported in the literature [49, 72].

### Late complications

Late complications occur after the acute post-operative period.

#### Electrode migration (Fig. 34)

An electrode array may migrate because of loss of support of the electrode lead, micro-movements in the soft tissue cover of the radical cavity, or a tension that pushes the electrode back [14]. Perimodiolar electrodes are affected less frequently and to a lower extent than lateral wall electrodes [73]. Minor migration may be asymptomatic or show a gradual increase in the impedance values in the basal electrodes [44, 74, 75]. Major back extrusion can lead to complete hearing loss.

**Fig. 34:**
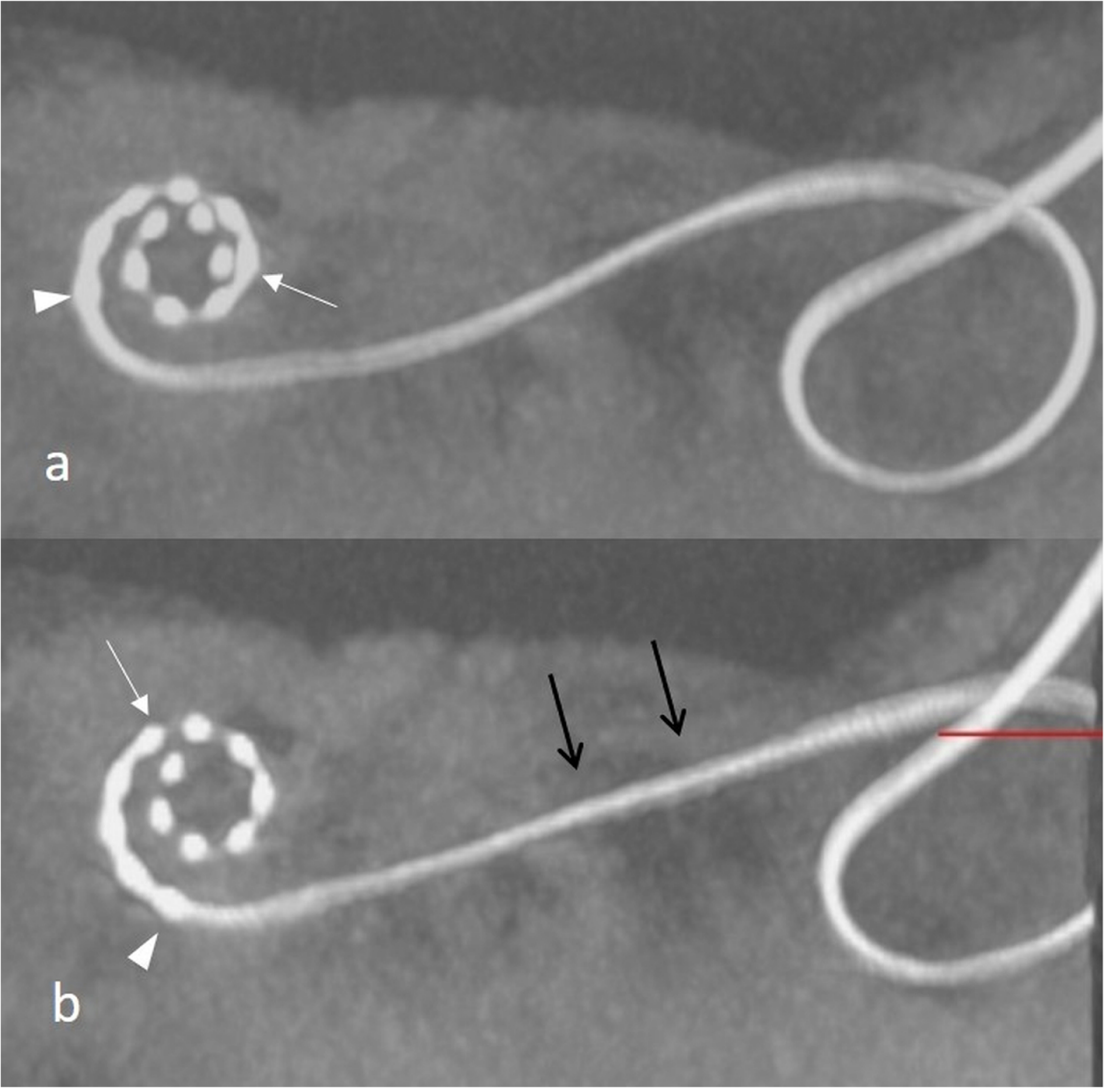
A 53-year-old male patient, with a complete malfunction of the CI and pain 3 months after CI surgery. CBCT paraaxial maximum intensity projection image shows initial overinsertion of the electrode array (**a**). The control scan shows back extrusion of the electrode array with migrated positions of the electrode tip (arrow) and basal electrode element (arrowhead) and straightened electrode array (black arrows) in the mastoidectomy cave (**b**)

#### Flap complications (Fig. 35)

Subcutaneous ulcer, infection, and wound dehiscence may occur at the implanted area of the magnet [14, 76]. Flap necrosis is the result of postsurgical malperfusion. Surgical revision is required. Local trauma may result in hematoma and magnet migration [77].

**Fig. 35:**
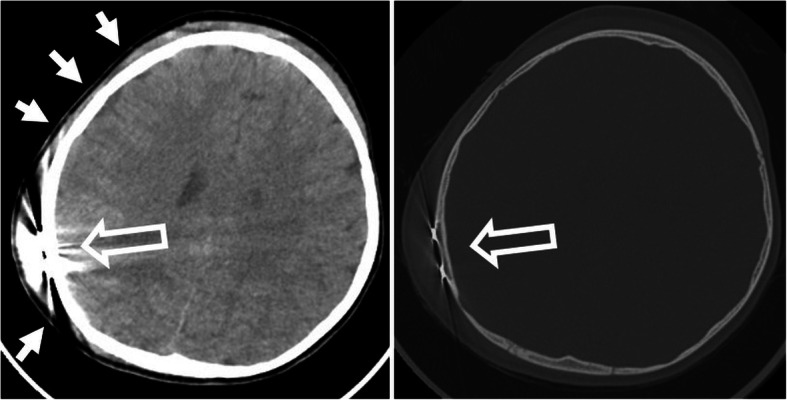
A 4-year-old male patient, with pain and insufficient hold of the speech processor after running into his brother. CT axial soft kernel image (left image) shows marked hematoma (arrows) in the skin at the frontal bone and temporal bone around the implanted magnet (hollow arrow). No intracranial bleeding. CT bone kernel axial image (right image) shows intact bone. The magnet is in the correct position

#### Bacterial labyrinthitis, otitis media, and cholesteatoma

Bacterial labyrinthitis may be secondary to the spread of middle ear flora into the cochlea. Otitis media and mastoiditis are more frequently observed in children. Cholesteatoma may occur after the inclusion of epithelial cells into the tympanic cavity [77, 78].

#### Fibrosis and delayed neural injury

Surgical trauma, foreign body tissue response, or disruption of any soft tissue or venous structure of scala tympani during insertion may induce intracochlear fibrosis [79]. The build-up of fibrosis and around the electrode over time will potentially impact or form a connection to the spiral ligament and basilar membrane, which will result in mechanical impedance with reduction or complete loss of hearing over time [80]. Delayed neural injury is explained by a molecular activation of apoptopic pathways by the insertion trauma and leads to continuous worsening of hearing [78].

## Conclusions

CI candidates need a thorough pre-operative imaging for diagnosis and classification of inner ear malformations and to identify any other abnormality in the temporal bone. HRCT and MRI are complementary and both image modalities are useful in patients with a history of meningitis, severe middle ear disease, and dysmorphic syndromes. Important contraindications such as aplasia and labyrinth sclerosis need to be ruled out. Implant surgeons need to be informed about any anatomical findings that may have an influence on the surgical procedure. After surgery, the position of the electrode can be evaluated using CBCT or HRCT. Scalar dislocation, cochlear dislocation, electrode fold, and malposition should be reported and may have important consequences for the patient, such as refined tuning or revision surgery.

## Data Availability

The data and image material were selected from the PACS-archive of our university hospital.

## Abbreviations

2TL: Two-turn length
3D-CISS: Three-dimensional constructive interference in steady-state
CBCT: Cone beam computed tomography
CDL: Cochlear duct length
CI: Cochlear implant
HRCT: High-resolution computed tomography
MRI: Magnetic resonance imaging
Non-EPI DWI: Non-echo-planar imaging diffusion-weighted imaging
SNHL: Sensorineural hearing loss
